# Nucleotide Combination Proportions Across Algae, Monocotyledons and Dicotyledons: Insights into Plant Genome Evolution

**DOI:** 10.3390/plants15172643

**Published:** 2026-08-28

**Authors:** Zhen Qin, Aixian Li, Yuanyuan Zhou, Zhicheng Jiang, Taifeng Du, Fuyun Hou

**Affiliations:** Crop Research Institute, Shandong Academy of Agricultural Sciences, Jinan 250100, China; qinzhenbio@163.com (Z.Q.); 13688628863@163.com (A.L.); zhou_yy_2020@163.com (Y.Z.); pyxjzc@163.com (Z.J.); a986947745@126.com (T.D.)

**Keywords:** comparative genomics, oligonucleotide composition, genome composition, nucleotide combination proportions, selection pressure

## Abstract

Plant evolution started with unicellular algae, gradually evolving multicellularity and terrestrial colonization. These evolutionary events were accompanied by the interplay of chromosome polyploidization, rearrangement, gene loss, and point mutation. We counted the proportion of nucleotide combinations in the genome sequences of 64 sequenced plants, and analyzed the significant difference in these nucleotide combination proportions among algae, monocotyledons and dicotyledons. The correlation of highly significant different and no significant different nucleotide combinations was analyzed respectively. Nucleotide combinations and their reverse complementary sequence proportions were analyzed in different functional regions of the genome. These results reveal that some nucleotide combinations are subject to strict selection, and these combinations have a higher proportion in the CDS regions and lower proportion in the intergenic regions. Meanwhile, there are some nucleotide combinations that are under less selective pressure, and these combinations have a higher proportion in the intergenic regions and lower proportion in the CDS regions. Cluster analysis based on trinucleotide to octanucleotide combination proportions reveals that plant genome evolution is accompanied by clade-wide differentiation of genome-wide nucleotide composition patterns, in addition to well-documented chromosomal polyploidization, structural rearrangement and gene loss events. We analyzed the changes in the proportion of nucleotide combinations at the genome level in 64 sequenced plants, providing a new idea for studying genome evolution in the plant kingdom.

## 1. Introduction

Plant evolution encompasses a remarkable transition from unicellular to multicellularity, from simple to complex organisms, and from aquatic to terrestrial life. Concomitantly, plant genome evolution is a complex and dynamic process driven by a multitude of molecular mechanisms. Point mutations, including base substitutions, insertions, and deletions, serve as the fundamental units of genomic variation. Their accumulation rate is influenced by various factors such as cell type, metabolic activity, and DNA repair efficiency [1]. Gene duplication events, whether tandem duplications of individual genes or duplications of larger genomic segments, provide critical evolutionary windows for generating genetic redundancy and novel functional traits [2]. Newly generated gene copies can gradually acquire unique biological functions through processes of subfunctionalization or neofunctionalization, leading to the expansion and increasing complexity of gene families [3].

Whole genome duplication (WGD), a large-scale polyploidization event, plays a particularly pivotal role in plant evolutionary history [4,5,6]. Polyploidization is of great significance for the formation of monocotyledon taxa. Combined with the fossil evidence and collinearity genes, it is presumed that the time of the evolutionary event of gramineous plants may have originated about 100 million years ago [7], and its ancestral species had a genome-wide doubling event [8]. This polyploidization had played an important role in promoting the generation of the new species of Gramineae and forming a large monocotyledonous plant family. Studies have shown that the common ancestor of core eudicots experienced a whole genome triplication event, also known as the gamma (γ) event, about 130 million years ago [9]. This event was confirmed in the genome structure analysis of Arabidopsis, apple, poplar and other species—that is, they all have the same hexaploidy ancestor [10,11]. Since then, polyploidy events have continued to occur in many plant lineages, such as the cotton genome [12], the Arabidopsis genome [13,14], and the soybean genome [15]. WGD not only results in a substantial increase in genome size but is also frequently accompanied by gene loss [16], chromosomal structural rearrangements [17], and the rewiring of gene regulatory networks [18]. These genomic alterations are pivotal in initiating isolation mechanisms and facilitating speciation. Furthermore, the active proliferation of transposable elements (TEs) is a significant contributor to genome evolution. The insertion, deletion, and recombination mediated by TEs not only contribute to variations in genome size but can also alter host gene expression patterns and even drive structural reorganization of the genome [19].

Hybridization events, particularly those leading to the formation of allopolyploids, are potent drivers of plant speciation and adaptive radiation. These events integrate genetic information from different parental genomes and are often followed by rapid genome stabilization processes [20].

The evolutionary rates of different gene families exhibit significant heterogeneity. Genes involved in essential metabolic and cellular functions generally display high degrees of conservation [21], while genes encoding transcription factors, signal transduction molecules, and those regulating environmentally relevant traits tend to undergo more rapid evolution [22]. Plants have also evolved sophisticated DNA repair mechanisms to maintain genomic stability [23], although the cumulative effect of background mutation rates remains foundational for long-term evolution.

We initially collected representative genomes from five major plant groups (algae, bryophytes, pteridophytes, monocotyledons, dicotyledons) to cover the full spectrum of plant terrestrialization and radiation. Consistent with our prior analyses on codon usage and SSR variation, bryophyte genomes display nucleotide composition profiles similar to dicotyledons, while pteridophytes resemble monocotyledons. To streamline cross-clade statistical comparison and focus on the three largest, well-sampled lineages with distinct evolutionary trajectories, we centered formal quantitative analyses on algae, monocotyledons and dicotyledons, while retaining bryophyte and pteridophyte species as auxiliary reference taxa throughout all clustering and proportion statistics. Our primary objective is to quantify the significant differences in these nucleotide combination proportions across these major plant groups and to correlate these variations with known evolutionary events.

By meticulously analyzing these nucleotide combination patterns at the genome level, this research intends to provide a novel perspective on plant genome evolution, offering a deeper insight into the interplay between mutation, selection, and genome structure that has driven the diversification of the plant kingdom. The findings are expected to advance our understanding of the fundamental molecular mechanisms underlying plant genome evolution and potentially inform future studies in comparative genomics and evolutionary biology.

## 2. Results

### 2.1. Information on 64 Sequenced Plant Genomes

**Figure 1:**
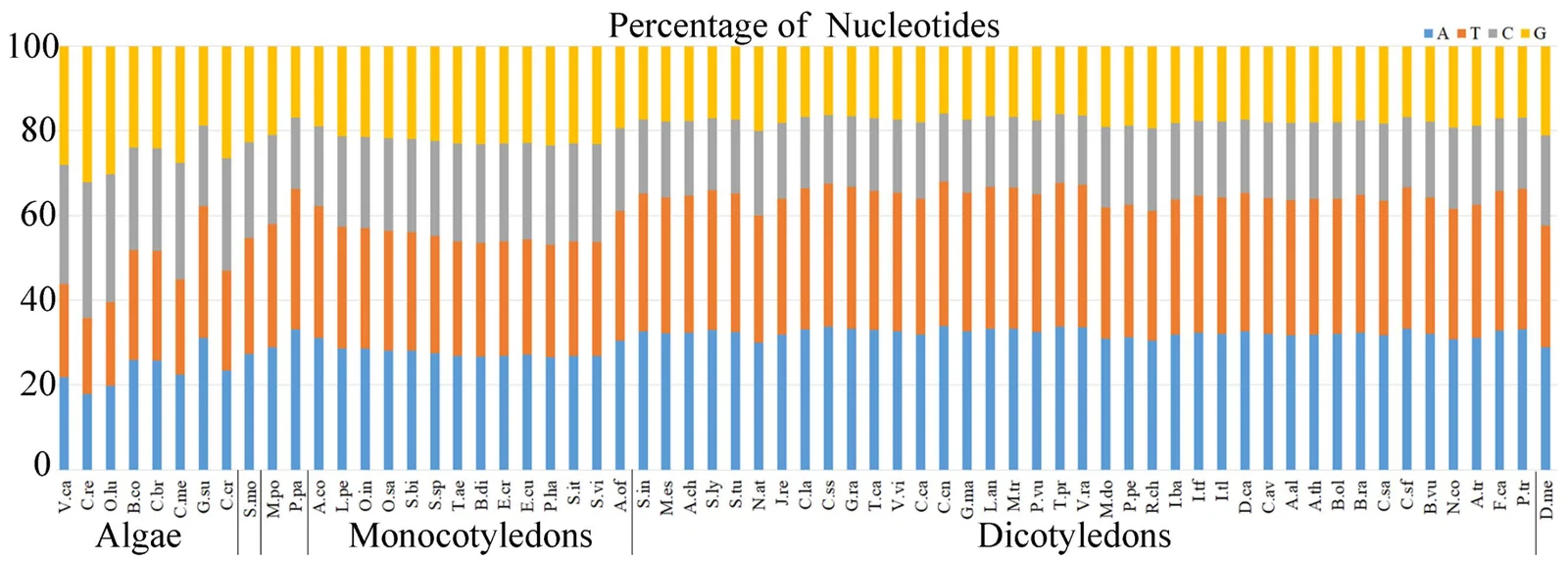
Proportion of mononucleotide in 64 plant genomes.

We have analyzed 64 sequenced plant genome sequences, with sizes ranging from 13 Mb to 14 Gb. Among them, the genomes of *Ostreococcus lucimarinus* (13.20 Mb), *Galdieria sulphuraria* (13.71 Mb), *Bathy coccus* (15.01 Mb), and *Cyanidioschyzon merolae* (16.55 Mb) are relatively small, with data volumes less than 100 Mb. The genomes of *Triticum aestivum* (14.07 Gb), *Saccharum spontaneum* (2.90 Gb), *Ipomoea batatas* (2.54 Gb), *Chara braunii* (1.45 Gb), *Echinochloa crusgalli* (1.29 Gb), and *Asparagus officinalis* (1.11 Gb) are larger, with data volumes exceeding 1 Gb. The remaining plant genomes range from 104.80 Mb to 949.18 Mb (Supplementary Table S1). We analyzed the mononucleotide proportions of 64 plant genome sequences and found that in the DNA single strands, the proportions of Adenine deoxyribonucleotide (A) and Thymine deoxyribonucleotide (T) are similar, while the proportions of Cytosine deoxyribonucleotide (C) and Guanine deoxyribonucleotide (G) are also similar. Based on the A&T proportions, plants can be clearly divided into three categories: algae, monocotyledons, and dicotyledons. Algal plants have the lowest A&T proportions, followed by monocotyledons, and dicotyledons have the highest A&T proportions. However, there are exceptions: *C. braunii* and *Bathy coccus*, which belong to the group of algae, have A&T proportions that are significantly higher than those of other algae but lower than those of monocotyledons; *G. sulphuraria*, also an algal plant, has an A&T proportion similar to that of dicotyledons. *Selaginella moellendorffii* belongs to the fern group, but its A&T proportion is similar to that of monocotyledons. *Physcomitrella patens* is a moss, but its A&T proportion is similar to that of dicotyledons. The A&T proportion of the monocotyledons *Ananas comosus* is significantly higher than that of other monocotyledons and is similar to that of dicotyledons (Figure 1 and Supplementary Table S2).

### 2.2. Analysis of Significant Differences in Nucleotide Combinations Proportion of Plant Genomes

We analyzed the proportion of different nucleotide combinations in algae, monocotyledons, and dicotyledons, ranging from dinucleotide to octanucleotide combinations. The percentages of combinations with highly significant differences (*p* ≤ 0.01) in proportion between algae and monocotyledons among these nucleotide combinations range from 15.63% to 19.89% (except for dinucleotide combinations, the same below). Similarly, the percentages of combinations with highly significant differences in proportion between algae and dicotyledons among these nucleotide combinations range from 41.41% to 56.25%. For combinations between monocotyledons and dicotyledons, the percentages of combinations with highly significant differences in proportion among these nucleotide combinations range from 72.31% to 88.28%. Among these nucleotide combinations, the percentages of combinations with highly significant differences in proportion across all three plant types range from 9.38% to 14.84% (Table 1 and Supplementary Table S3).

The percentages of combinations with no significant differences in proportion between algae and monocotyledons, as well as between algae and dicotyledons, account for 57.32–68.75% and 21.88–35.59% of the respective nucleotide combinations, respectively. Similarly, the percentages of combinations with no significant differences in proportion between monocotyledons and dicotyledons account for 7.42–20.27% of the nucleotide combinations. For combinations involving algae, monocotyledons, and dicotyledons, the percentages of combinations with no significant differences in proportion across the three plant types range from 5.86 to 10.56% (Table 1 and Supplementary Table S3).

**Figure 2:**
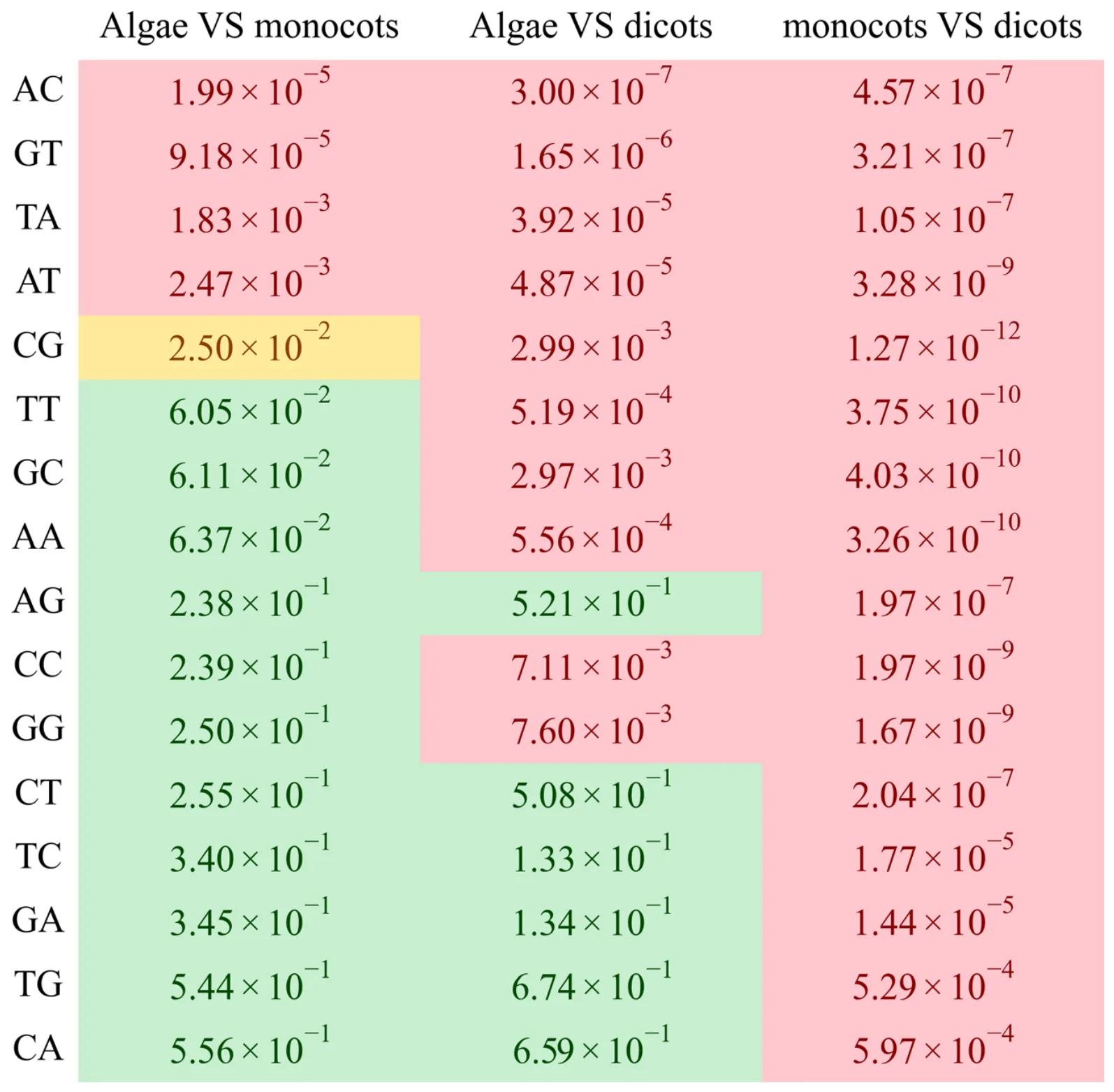
Significance analysis of dinucleotide combination proportions among species. Red represents highly significant differences (*t*-test, *p* ≤ 0.01), yellow represents significant differences (*t*-test, *p* < 0.05), and green represents no significant differences (*t*-test, *p* > 0.05).

Based on the analysis of species according to the proportion of dinucleotide combinations, there are four nucleotide combinations (AC/GT, TA/AT) with highly significant differences (*t*-test, *p* ≤ 0.01) between algae, monocotyledons, and dicotyledons, accounting for 25% of the total number of two nucleotide combinations. CG/GC, AA/TT, and CC/GG showed highly significant differences (*t*-test, *p* ≤ 0.01) between algae and dicotyledons. No nucleotide combination proportion shows no significant differences (*t*-test, *p* > 0.05) among algae, monocotyledons, and dicotyledons (Figure 2 and Supplementary Table S4).

**Figure 3:**
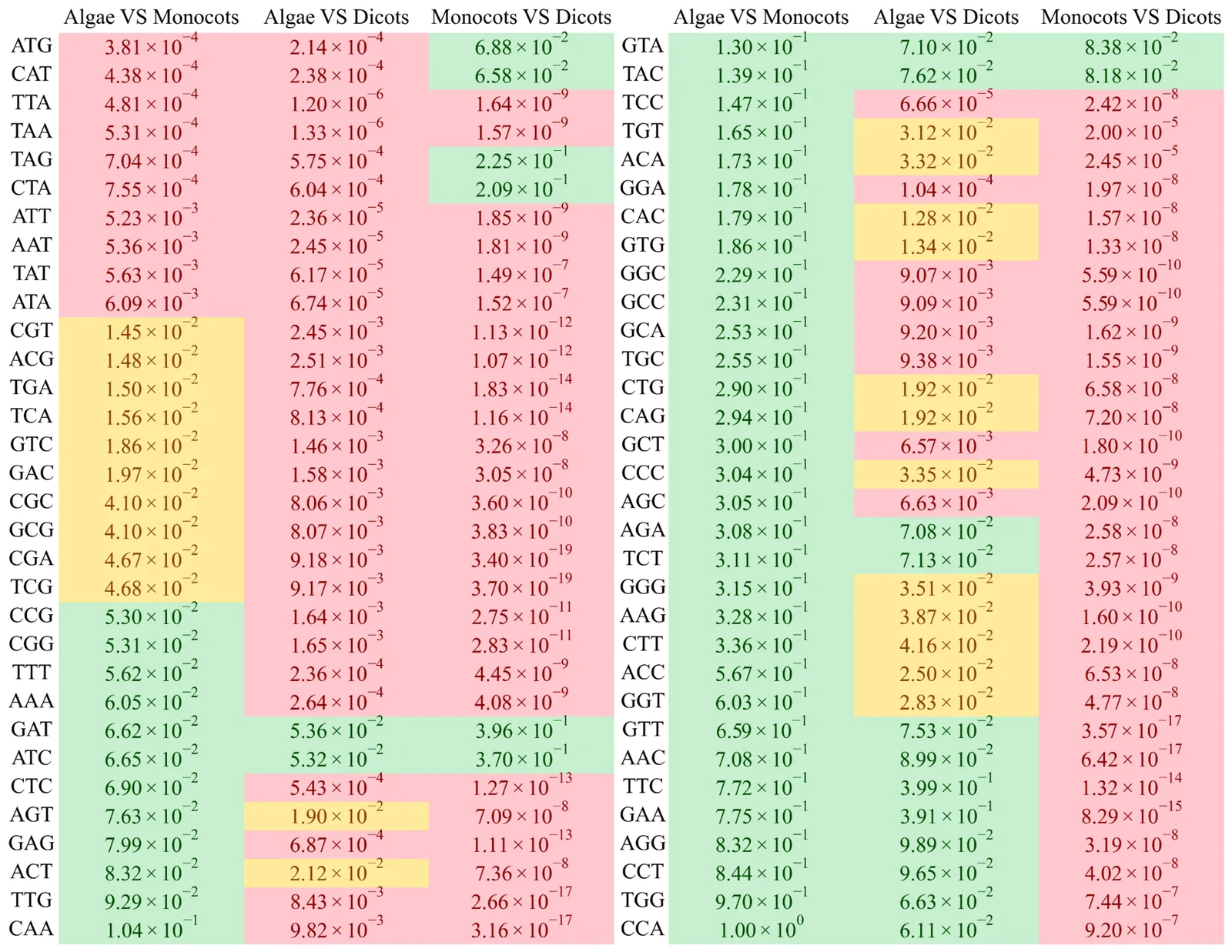
Significance analysis of trinucleotide combination proportions among species. Red represents highly significant differences (*t*-test, *p* ≤ 0.01), yellow represents significant differences (*t*-test, *p* < 0.05), and green represents no significant differences (*t*-test, *p* > 0.05).

Based on the analysis of species according to the proportion of trinucleotide combinations, there are highly significant differences in the distribution of TAA/TTA, AAT/ATT, and ATA/TAT combinations among the three types of plants (*t*-test, *p* ≤ 0.01). Significant differences are also observed in the distribution of CGT/ACG, TGA/TCA, GTC/GAC, CGC/GCG, and CGA/TCG among the three types of plants (*t*-test, *p* < 0.05). However, no significant differences are found in the proportion of ATC/GAT and GTA/TAC among the three types of plants (*t*-test, *p* > 0.05). Except for ATG/CAT, TAG/CTA, GAT/ATC, and GTA/TAC, all other trinucleotide combinations show highly significant differences between monocotyledonous and dicotyledonous plants (*t*-test, *p* ≤ 0.01) (Figure 3 and Supplementary Table S5).

Based on the analysis of species according to the proportion of tetranucleotide combinations, 38 nucleotide combinations show highly significant differences (*t*-test, *p* ≤ 0.01), and 15 nucleotide combinations show no significant differences among algae, monocotyledons, and dicotyledons (*t*-test, *p* > 0.05) (Table 1), accounting for 14.84% and 5.86% of the total number of tetranucleotide combinations, respectively (Supplementary Table S6).

Based on the analysis of species according to the proportion of pentanucleotide combinations, 142 nucleotide combinations show highly significant differences (*t*-test, *p* ≤ 0.01), and 62 nucleotide combinations show no significant differences among algae, monocotyledons, and dicotyledons (*t*-test, *p* > 0.05) (Table 1), accounting for 13.87% and 6.05% of the total number of pentanucleotide combinations, respectively (Supplementary Table S7).

Based on the analysis of species according to the proportion of hexanucleotide combinations, 558 nucleotide combinations show highly significant differences (*t*-test, *p* ≤ 0.01), and 276 nucleotide combinations show no significant differences among algae, monocotyledons, and dicotyledons (*t*-test, *p* > 0.05) (Table 1), accounting for 13.62% and 6.74% of the total number of hexanucleotide combinations, respectively (Supplementary Table S8).

Based on the analysis of species according to the proportion of heptanucleotide combinations, 2130 nucleotide combinations show highly significant differences (*t*-test, *p* ≤ 0.01), and 1304 nucleotide combinations show no significant differences among algae, monocotyledons, and dicotyledons (*t*-test, *p* > 0.05) (Table 1), accounting for 13.00% and 7.96% of the total number of heptanucleotide combinations, respectively (Supplementary Table S9).

Based on the analysis of species according to the proportion of octanucleotide combinations, 7317 nucleotide combinations show highly significant differences (*t*-test, *p* ≤ 0.01), and 6918 nucleotide combinations show no significant differences among algae, monocotyledons, and dicotyledons (*t*-test, *p* > 0.05) (Table 1), accounting for 11.16% and 10.56% of the total number of octanucleotide combinations, respectively (Supplementary Table S10).

**Figure 4:**
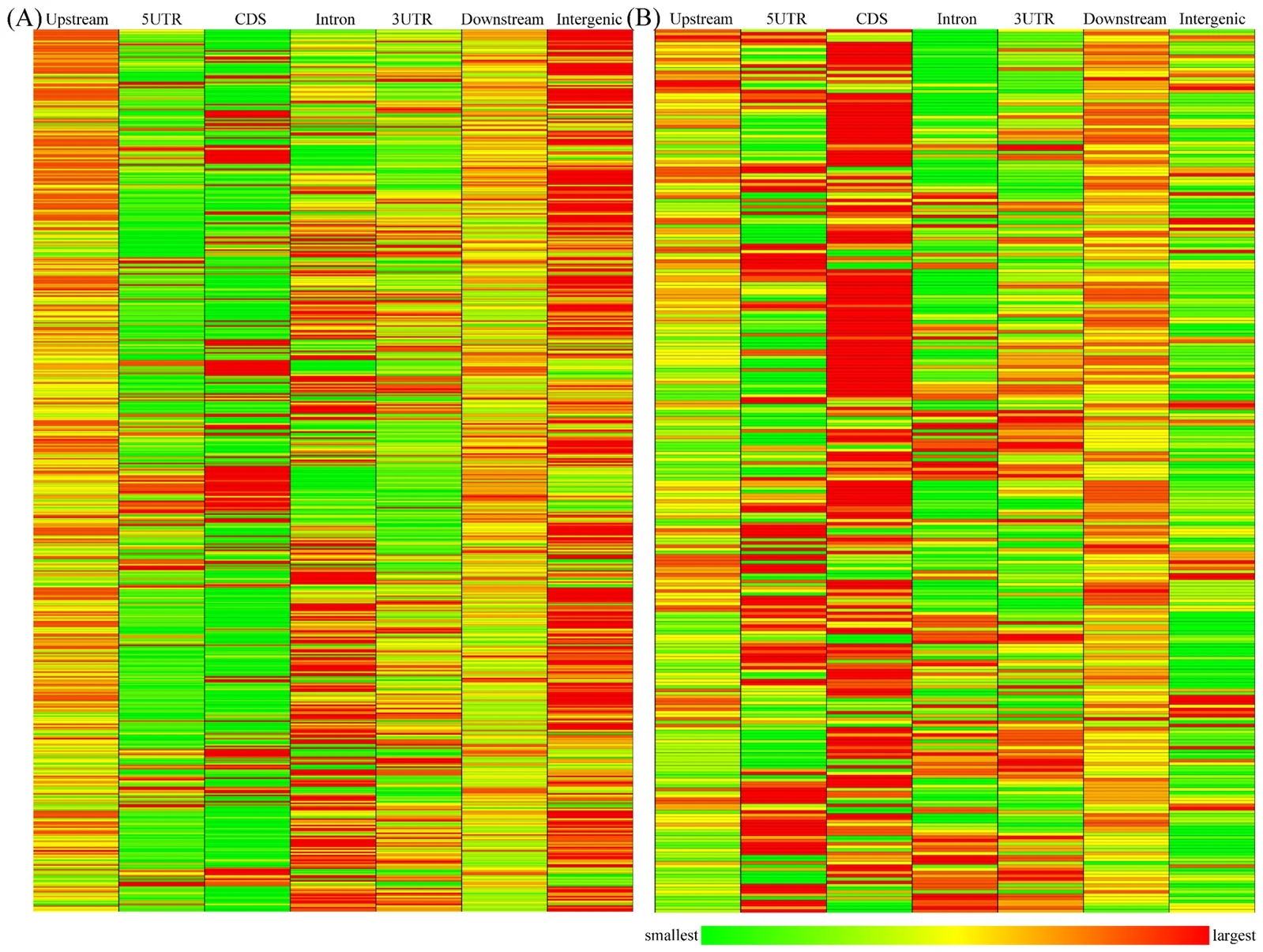
Heatmap of hexanucleotide combinations proportions. (**A**). Heatmap of hexanucleotide combinations proportions with highly significant differences among species. (**B**). Heatmap of hexanucleotide combinations proportions with no significant differences among species. Each line has seven values, with the largest value in red and the smallest in green.

We used the Arabidopsis genome sequence to analyze the proportion of highly significant-differences and no-significant-differences nucleotide combinations on the gene upstream 1000 bp, 5′UTR, CDS, intron, 3′UTR, gene downstream 1000 bp, and intergenic region in order to reveal their relationship with evolution. The results show that the nucleotide combinations with highly significant differences have higher proportions in intergenic and intron regions than other regions, and lower proportions in CDS and 5′UTR regions than other regions (Figure 4A and Supplementary Table S11). Nucleotide combinations with no significant differences have a higher proportion in CDS regions and a lower proportion in intergenic regions (Figure 4B and Supplementary Table S11). There is no preference for the distribution of highly significant- and no-significant-differences nucleotide combinations in the upstream and downstream regions (Figure 4 and Supplementary Table S11).

### 2.3. Correlation Analysis of Nucleotide Combinations with Highly Significant Differences

**Figure 5:**
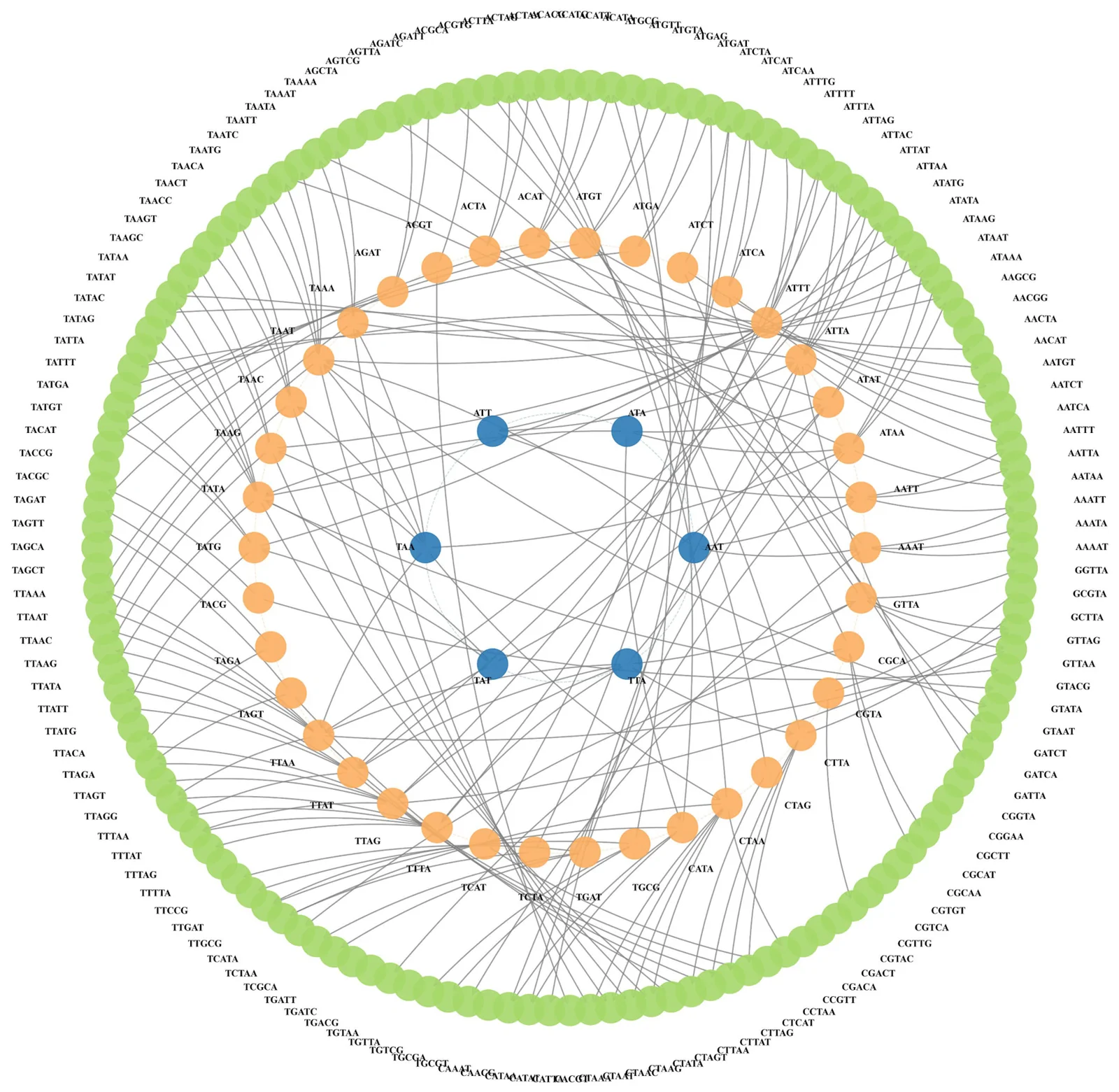
Correlation analysis of nucleotide combinations with highly significant differences. Two nucleotide combinations with inclusion relationships are linked by lines. Blue, orange, and green denote trinucleotide, tetranucleotide, and pentanucleotide combinations, respectively.

We analyzed the nucleotide combinations that exhibit highly significant differences among algae, monocotyledons, and dicotyledons. There is a total of 34 types of trinucleotide combinations included in the tetranucleotide combinations. Among them, six nucleotide combinations match the trinucleotide combinations with highly significant differences, accounting for 100% of the latter’s total. The total type number of tetranucleotide combinations included in the pentanucleotide combination is 118, of which 38 are consistent with the highly significant-difference tetranucleotide combination, accounting for 100% of the highly significant-difference tetranucleotide combination (Figure 5, Table 2 and Supplementary Table S3). The total type number of pentanucleotide combinations included in the hexanucleotide combination is 450, of which 138 nucleotide combinations are consistent with the highly significant-difference pentanucleotide combination, accounting for 95.83% of the highly significant-difference pentanucleotide combination. The total type number of hexanucleotide combinations included in the heptanucleotide combination is 1697, of which 565 nucleotide combinations are consistent with the highly significant-difference hexanucleotide combination, accounting for 97.92% of the highly significant-difference hexanucleotide combination (Table 2 and Supplementary Table S3).

### 2.4. Correlation Analysis of Nucleotide Combinations with No Significant Difference

**Figure 6:**
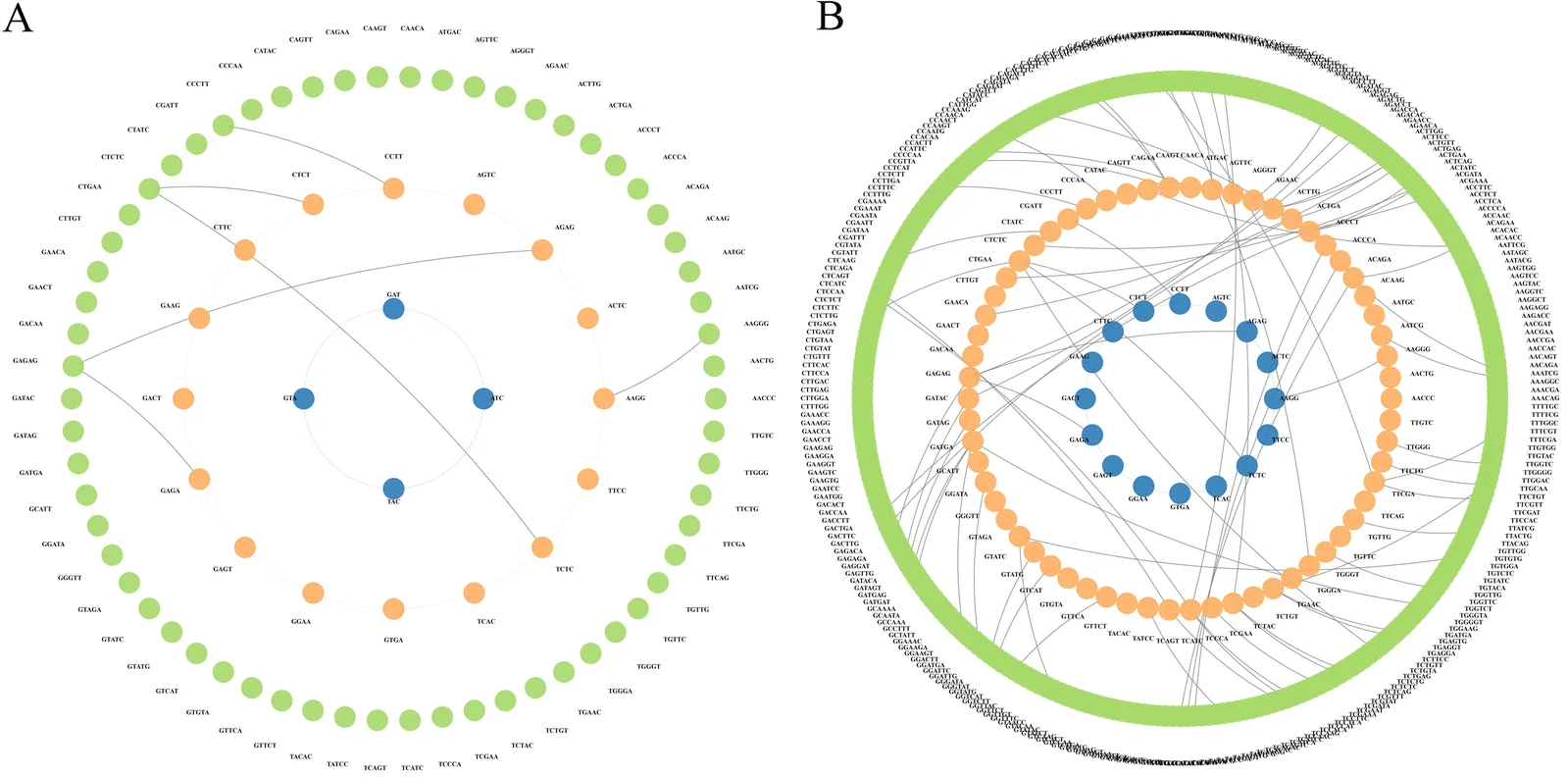
Correlation analysis of nucleotide combinations with no significant difference. (**A**). Correlation analysis of trinucleotide to pentanucleotide combinations with no significant difference. Blue, orange, and green denote trinucleotide, tetranucleotide, and pentanucleotide combinations, respectively. (**B**). Correlation analysis of tetranucleotide to hexanucleotide combinations with no significant difference. Two nucleotide combinations with inclusion relationships are linked by lines. Blue, orange, and green denote tetranucleotide, pentanucleotide, and hexanucleotide combinations, respectively.

We analyzed the nucleotide combinations that exhibit no significant differences among algae, monocotyledons, and dicotyledons. There is a total of 20 types of trinucleotide combinations included in the tetranucleotide combinations. Among them, zero nucleotide combinations matched the trinucleotide combinations with no significant differences, accounting for 0% of the latter’s total. The total type number of tetranucleotide combinations included in the pentanucleotide combination is 81, of which six are consistent with the no-significant-difference tetranucleotide combination, accounting for 37.50% of the no-significant-difference tetranucleotide combination. The total type number of pentanucleotide combinations included in the hexanucleotide combination is 328, of which 42 nucleotide combinations are consistent with the no-significant-difference pentanucleotide combination, accounting for 67.74% of the no-significant-difference pentanucleotide combination. The total type number of hexanucleotide combinations included in the heptanucleotide combination is 1514, of which 233 nucleotide combinations are consistent with the no-significant-difference hexanucleotide combination, accounting for 81.47% of the no-significant-difference hexanucleotide combination (Figure 6, Table 3 and Supplementary Table S3).

### 2.5. Analysis of Nucleotide Combination and Its Reverse Complementary Sequence

**Figure 7:**
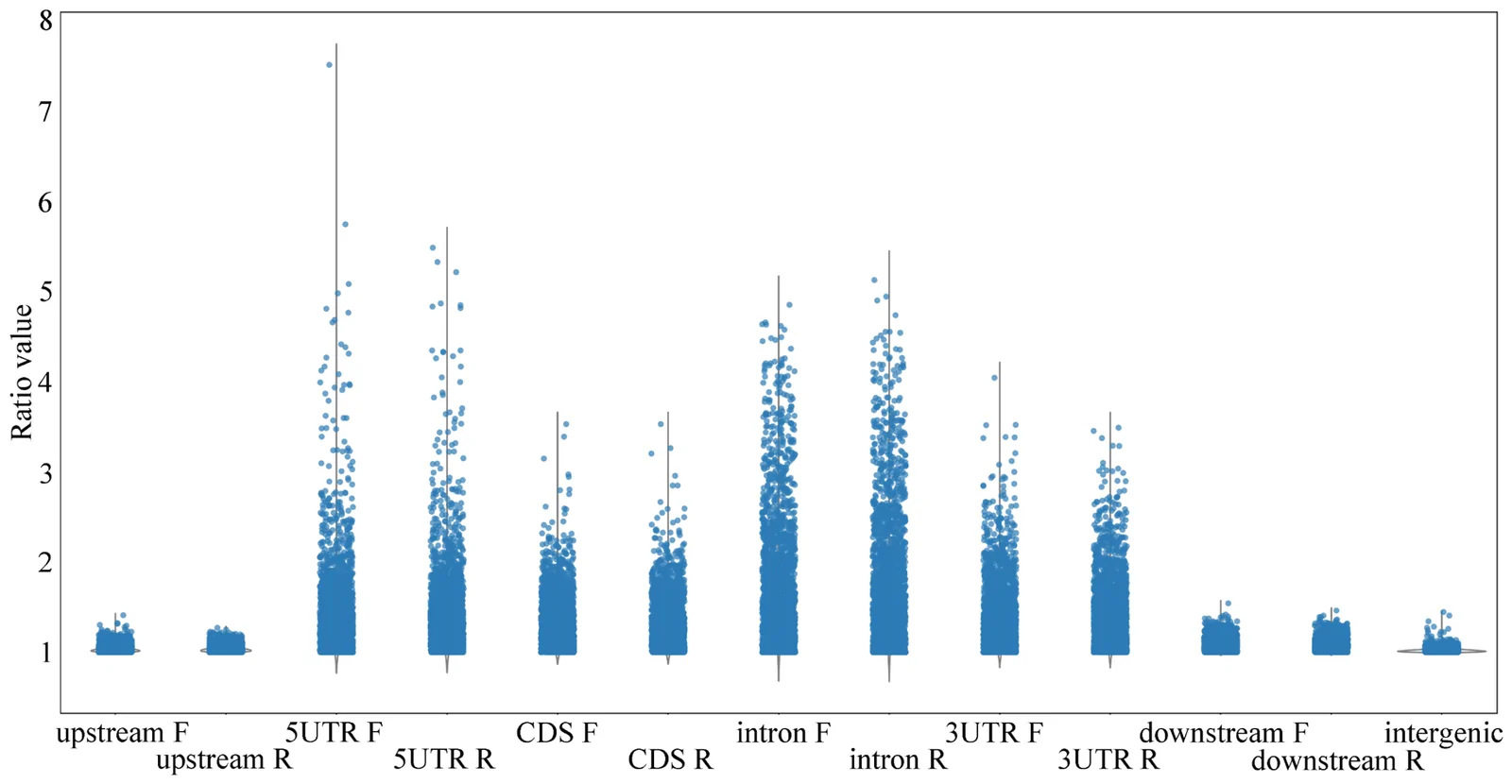
Analysis of *Arabidopsis thaliana* hexanucleotide combinations and their reverse complementary sequences.

We know that chromosome exists in the form of DNA double helices through base complementary pairing, where the two strands of chromosomes complement each other in reverse [24]. We analyzed the proportion of different nucleotide combinations on a single strand of 64 plant chromosomes and found that from the perspective of plant genomes, the proportion of mono-octa nucleotide combinations to their reverse complementary sequences is similar and smaller than the difference in the proportion between different nucleotide combinations (i.e., non-reverse complementary sequences) (Supplementary S1). In order to further analyze the reasons for the similar proportion of reverse complementary sequences, we divided *Arabidopsis thaliana* chromosomes into seven categories: CDS region, intron region, 5′UTR region, 3′UTR region, gene upstream 1000 bp region, gene downstream 1000 bp region, and gene intergenic sequence for analysis. It is found that some nucleotide combinations in CDS region, intron region, 5′UTR region, and 3′UTR region are not similar to their proportion of reverse complementary sequences, while nucleotide combinations in gene upstream 1000 bp, gene downstream 1000 bp, and gene intergenic region are similar to their proportion of reverse complementary sequences. We further analyzed the sense chain and antisense chain separately and found that the different nucleotide combination proportions within the CDS, intron, 5′UTR, and 3′UTR regions remain consistent between the sense chain and antisense chain (Figure 7 and Supplementary Table S12). In this way, the corresponding sequences on the sense chain corresponding to each region of the gene located on the antisense chain, namely the reverse complementary sequences, remain consistent in proportion. The distribution of genes (number and length) on the two strands of a chromosome (sense strand and antisense strand) is similar, so analyzing different nucleotide combinations and their reverse complementary sequence proportions from the perspective of a single strand of a chromosome (such as the sense strand) will maintain similarity.

Figure 7 shows the proportion of hexanucleotide combinations of seven regions in the sense and antisense of the *Arabidopsis thaliana* genome, including CDS, intron, 5′UTR, 3′UTR, upstream 1000 bp of the gene, downstream 1000 bp of the gene, and intergenic sequence regions. Each point in the figure represents the ratio of nucleotide combinations to the number of their reverse complementary sequences.

As a control, we randomly generated a 20 Mb nucleotide sequence with a Perl program. Analysis of nucleotide combination proportion revealed that different nucleotide combinations with the same number of nucleotides have similar proportions and no significant differences (Supplementary S2).

### 2.6. Evolution Analysis of Plant Genomes

Based on the analysis of the combination proportions of trinucleotide to octanucleotide, algae, monocotyledons, and dicotyledons can be clustered separately (Supplementary Figure S1). Cluster analysis revealed that *C. merolae*, *Chondrus crispus*, *Volvox carteri*, *Chlamydomonas reinhardtii*, *Ostreococcus lucimarinus*, and *Bathy coccus* clustered together, while *C. braunii* was more closely related to monocotyledonous plants than to the above species. Cluster analysis revealed that 12 out of the 14 monocotyledonous plants analyzed clustered together. Species belonging to the same genus are clustered together, such as *Setaria italica* and *Setaria viridis*, and *Oryza sativa indica* and *Oryza sativa japonica*.

## 3. Discussion

In this study, algae, bryophyte, pteridophyta, monocotyledons and dicotyledons were initially selected for analysis, which largely cover the main lineage of plant evolution. Combining the findings from our previous studies based on codon usage frequency [25] and SSR [26] with the results obtained in the present work, we observed that bryophytes share similar genomic features with dicotyledons, whereas the genomes of pteridophyta are more analogous to those of monocotyledons. To dissect the universal rules of plant evolution, we further chose algae, monocotyledons and dicotyledons as research subjects, aiming to explore the fundamental genetic characteristics during plant evolutionary processes.

Based on the analysis of the proportion of nucleotide combinations in 64 plant genomes, we find highly significant-differences nucleotide combinations and no-significant-differences nucleotide combinations among algae, monocotyledons, and dicotyledons. The proportion of highly significant-differences nucleotide combinations in intergenic and intron regions is higher, while the proportion in CDS and 5′UTR regions is lower. Nucleotide combinations with highly significant differences among species imply that these combinations are under less selective pressure in the process of evolution and may be the basis of species diversity. The proportion of no-significant-differences nucleotides in CDS regions is higher, and the proportion in intergenic regions is lower. Nucleotide combinations with no significant differences among species imply that they have been under greater selection pressure in the process of evolution. This corresponds to their distribution proportions in the genome’s different regions. These results show that the selection pressure on CDS regions is significantly higher than that on other regions, and the selection pressure on intergenic sequences is significantly lower than that on other regions.

Compared with highly significant-difference nucleotide combinations, the percentages of matching the next level nucleotide combinations in no-significant-difference nucleotide combinations is lower. It is speculated that these nucleotide combinations with no significant differences may be components of certain important structures (such as protein domain or cis-acting element), which vary relatively little between species due to significant selection pressure.

On the whole level of the genome, the proportion of nucleotide combinations and their reverse complementary sequences is similar, but the proportion is different in different regions, indicating that different regions are subject to different selection pressures. The proportion of nucleotide combinations and their reverse complementary sequences in the CDSs, introns, 5′UTR, and 3′UTR regions of the gene varies greatly, indicating that these regions are subjected to significantly higher selection pressure. The proportion in the upstream 1000 bp, downstream 1000 bp, and intergenic sequence regions of genes differed less, indicating that these regions are subjected to lower selection pressure. The results also support that the selection pressure on CDS regions is significantly greater than that on intergenic sequence regions.

Analysis of the randomly generated nucleotide combination proportion revealed that different nucleotide combinations with the same number of nucleotides have similar proportions and no significant differences. This also confirms that the different nucleotide combination proportion in different regions of the gene is the result of selection pressure.

*I. batatas* is a hexaploid, and it is clustered together with two diploid species belonging to the same genus, *Ipomoea trifida* and *Ipomoea triloba*. *I. batatas* is located on the periphery of *I. trifida* and *I. triloba*, indicating that the increase in ploidy provides a basis for changes in nucleotide composition proportion [27,28]. The results indicate that evolutionary analysis based on nucleotide combination proportion is scientific. The evolution of plant genomes involves not only the replication [29], recombination, and loss of chromosomal segments [30], but also at the level of nucleotide combination across the entire genome. The analysis based on genetic codons [25] and SSR [26] also supports that plant evolution is an overall evolution of the entire genome nucleotide.

*C. braunii* belongs to Charophytic algae, which are the closest living relatives of land plants. From the perspective of k-mer proportion analysis of tri-octa nucleotide combinations, the genome of *C. braunii* is found to be closer to that of land plants. This indicates that the evolution of *C. braunii* involves not only functional adaptations [31] but also the evolution of the entire genomic nucleotide composition.

It is well established that plant evolution has proceeded over hundreds of millions of years, accompanied by abundant genomic evolutionary events. Diverse plant species and lineages have evolved unique morphological traits, which are matched by lineage-specific genomic signatures. During long-term plant evolution, genomic sequences and structures are constantly subject to variation, while core genomic framework features remain relatively conserved. Since genotype determines phenotype, genomic architecture is intrinsically linked to plant phenotypic characteristics. Accordingly, we propose a working hypothesis that extant plant genomes generally retain core nucleotide-composition signatures inherited from ancestral plant genomes. We explicitly acknowledge that this premise remains unvalidated by direct evidence and carries multiple inherent limitations when inferring macroevolutionary patterns across deeply divergent plant lineages: First, modern plant genomes have undergone billions of years of lineage-specific genomic remodeling, including repeated whole-genome duplications, massive gene loss, transposon burst, horizontal gene transfer, and lineage-specific point mutation accumulation. These post-speciation genomic rearrangements inevitably overwrite partial ancestral nucleotide signatures, weakening the resolution for deep-time evolutionary inference. Second, our analysis relies solely on nucleotide combination proportions from contemporary sequenced species; we lack fossil genomic evidence to calibrate how k-mer composition shifts along geological timescales. Divergence time estimates between algae, monocots and dicots span hundreds of millions of years, and single-layer nucleotide composition data cannot fully disentangle the confounding effects of neutral drift and directional natural selection. Third, uneven taxonomic sampling across early-branching clades (algae, bryophytes, pteridophytes) restricts our ability to distinguish universal ancestral patterns from clade-specific derived genomic features. Despite these constraints, consistent differentiation of trinucleotide–octanucleotide proportions between algae, monocots and dicots still provides a novel comparative perspective to interpret broad-scale plant genome evolution, complementary to traditional evidence from collinearity, gene families and codon usage bias. Particular species can experience distinctive genomic alterations driven by environmental adaptation or polyploidization, such as *G. sulphuraria*, *A. comosus* and *C. braunii*, which show deviant A/T content and nucleotide composition clustering.

*G. sulphuraria*, as a red alga, does not cluster with other algae in the tri-hepta nucleotide combination proportion analysis; instead, it clusters more closely with dicotyledonous plants. Existing research suggests that the environmental adaptability of *G. sulphuraria* appears to be achieved through horizontal gene transfer from various bacteria and archaea. These horizontally transferred genes often undergo expansion within gene families, further enhancing their adaptability. At least 5% of the protein-coding genes in *G. sulphuraria* may have been acquired through horizontal gene transfer [32].

*A. comosus*, a member of the Poales order Bromeliaceae family, is not grouped together with the Poaceae family, which also belongs to the Poales order. The pan-cereal genome duplication event (ρ) occurred before the divergence of lineages leading to rice and wheat. However, this event postdates the divergence for the lineages leading to the Poaceae family and Bromeliaceae family (*A. comosus*) [33]. After ρ duplication, the Poaceae family genome underwent large-scale gene loss and chromosomal rearrangement [34]. The proportions of combinations of tri-octa nucleotides also support the notion that the Poaceae and Bromeliaceae underwent distinct evolutions after their divergence. This difference is not only present at the chromosomal level, but also at the nucleotide combination level.

## 4. Materials and Methods

### 4.1. Plant Species Information

In this paper, 64 sequenced plant genomes were selected for comparative genomic analysis, together with *Drosophila melanogaster* as an outgroup control (65 taxa in total, Supplementary Table S1). The species selection followed three strict criteria: (1) high-quality chromosome-level or scaffold-level published genome assemblies with complete gene functional annotations; low-contiguity draft genomes containing massive unknown N-base gaps were excluded to avoid biased k-mer counting results; (2) phylogenetic representativeness: we prioritized species covering all major plant evolutionary clades, including algae, bryophytes, pteridophytes, monocotyledons and dicotyledons, to capture broad variation in nucleotide composition across the plant tree of life; (3) data accessibility: all genome FASTA files were downloaded from public databases with open access for secondary bioinformatic mining.

### 4.2. Nucleotide Combinations Proportion Statistics

We had calculated the proportion of nucleotide combinations consisting of mononucleotide to octanucleotide on plant genome sequences. The principle of genomic nucleotide combinations statistics was based on one two three, two three four and three four five, etc., with Perl script (Supplementary S3). The proportion of a nucleotide combination referred to the proportion of the total number of occurrences of a specific nucleotide combination (e.g., AAA) in a plant to the total number of such types of nucleotide combinations (NNN) in this plant. For example, in the *Arabidopsis thaliana* genome, the combination AAA appears a total of 5,250,461 times, and the combination of trinucleotides appears a total of 118,959,501 times. Therefore, the proportion of the AAA combination was 5,250,461/118,959,501. For different chromosomes within the same species, the proportion of nucleotide combinations was calculated by individually counting the number of occurrences of each combination, adding up the counts for the same nucleotide combination across different chromosomes, and then calculating the proportion. For the unsequenced regions on the genome, that was, the occurrence of N/n in the genome sequence, the nucleotide combinations containing N/n were not within the scope of this study.

### 4.3. Analysis of Significant Differences in Nucleotide Combinations Proportion of Plant Genomes

The eight algae, fourteen monocotyledons, and thirty-nine dicotyledons analyzed were categorized into three groups according to the evolutionary relationship. The proportion of the same nucleotide combinations in these three groups was analyzed using the *t*-test method. A two-sample *t*-test assuming unequal variances and a two-tailed test were conducted. Based on a *p*-value, a smaller *p*-value indicates a greater difference in the distribution of nucleotide combinations between the two compared groups. Dinucleotide and octanucleotide combinations were analyzed separately.

### 4.4. Analysis of Nucleotide Combinations and Its Reverse Complementary Sequences

We calculated the absolute value of the proportion difference between two different nucleotide combinations with the same number of nucleotides respectively, and added up the absolute values of the differences in the same combination of 64 plant genomes. The larger the sum, the greater the difference in the proportion between two nucleotide combinations, and the smaller the sum, the smaller the difference in the proportion between two nucleotide combinations.

### 4.5. Clustering Analysis Based on the Proportion of Nucleotide Combinations

Symmetrized Kullback–Leibler divergence analysis [35], an amount measuring the difference between *p* and *q* of two species, was defined as

$$\sum_{x}\left(p\left(x\right)\times log\frac{p\left(x\right)}{q\left(x\right)}+q\left(x\right)\times log\frac{q(x)}{p(x)}\right)\times \frac{1}{2}$$

according to the proportion of trinucleotide combination to octanucleotide combinations, *p*(*x*) and *q*(*x*) represent the proportion of the same nucleotide composition in the two species, respectively, and *x* represents different nucleotide combinations with the same number of nucleotides in the two species. All pairwise comparisons (including controls) between 65 genomes were performed. According to the symmetrized Kullback–Leibler divergence analysis, the UPGMA method of MEGA4 software package was used for cluster analysis.

## Figures and Tables

**Table 1:** Significance analysis of nucleotide combination proportions among species.

**Algae vs. Monocots**	**Number (≤0.01)**	**Number (>0.05)**	**Total number of combinations**	**% (** **≤** **0.01)**	**% (>0.05)**
Dinucleotide combinations	4	11	16	25.00	68.75
Trinucleotide combinations	10	44	64	15.63	68.75
Tetranucleotide combinations	44	173	256	17.19	67.58
Pentanucleotide combinations	192	587	1024	18.75	57.32
Hexanucleotide combinations	786	2451	4096	19.19	59.84
Heptanucleotide combinations	3259	9851	16,384	19.89	60.13
Octanucleotide combinations	12,736	40,484	65,536	19.43	61.77
**Algae vs. Dicots**	**Number (** **≤** **0.01)**	**Number (>0.05)**	**Total number of combinations**	**% (** **≤** **0.01)**	**% (>0.05)**
Dinucleotide combinations	10	6	16	62.50	37.50
Trinucleotide combinations	36	14	64	56.25	21.88
Tetranucleotide combinations	117	69	256	45.70	26.95
Pentanucleotide combinations	508	284	1024	49.61	27.73
Hexanucleotide combinations	1906	1336	4096	46.53	32.62
Heptanucleotide combinations	7302	5364	16,384	44.57	32.74
Octanucleotide combinations	27,136	23,323	65,536	41.41	35.59
**Monocots vs. Dicots**	**Number (** **≤** **0.01)**	**Number (>0.05)**	**Total number of combinations**	**% (** **≤** **0.01)**	**% (>0.05)**
Dinucleotide combinations	16	0	16	100.00	0.00
Trinucleotide combinations	56	8	64	87.50	12.50
Tetranucleotide combinations	226	19	256	88.28	7.42
Pentanucleotide combinations	869	122	1024	84.86	11.91
Hexanucleotide combinations	3437	463	4096	83.91	11.30
Heptanucleotide combinations	12,996	2493	16,384	79.32	15.22
Octanucleotide combinations	47,389	13,281	65,536	72.31	20.27
**Algae vs. Monocots vs. Dicots**	**Number (** **≤** **0.01)**	**Number (>0.05)**	**Total number of combinations**	**% (** **≤** **0.01)**	**% (>0.05)**
Dinucleotide combinations	4	0	16	25.00	0.00
Trinucleotide combinations	6	4	64	9.38	6.25
Tetranucleotide combinations	38	15	256	14.84	5.86
Pentanucleotide combinations	142	62	1024	13.87	6.05
Hexanucleotide combinations	558	276	4096	13.62	6.74
Heptanucleotide combinations	2130	1304	16,384	13.00	7.96
Octanucleotide combinations	7317	6918	65,536	11.16	10.56

**Table 2:** Nucleotide combinations with highly significant differences (≤0.01).

Nucleotide Combinations with Highly Significant Differences (≤0.01)	Num	Total Num	%
The types of trinucleotide combinations contained within tetranucleotide combinations	34		
The number of nucleotide combinations that are consistent with trinucleotide combinations	6	6	100
The number of nucleotide combinations that are inconsistent with the trinucleotide combinations	28		
The types of tetranucleotide combinations contained within pentanucleotide combinations	118		
The number of nucleotide combinations that are consistent with tetranucleotide combinations	38	38	100
The number of nucleotide combinations that are inconsistent with the tetranucleotide combinations	80		
The types of pentanucleotide combinations contained within hexanucleotide combinations	450		
The number of nucleotide combinations that are consistent with pentanucleotide combinations	138	144 *	95.83
The number of nucleotide combinations that are inconsistent with the pentanucleotide combinations	312		
The types of hexanucleotide combinations contained within heptanucleotide combinations	1697		
The number of nucleotide combinations that are consistent with hexanucleotide combinations	565	577 *	97.92
The number of nucleotide combinations that are inconsistent with the hexanucleotide combinations	1132		

* Represents nucleotide combinations with highly significant differences, and their reverse complementary sequence combinations which only exhibit significant differences. The *p*-values for these combinations are only slightly higher than 0.01.

**Table 3:** Correlation analysis of nucleotide combinations with no significant difference.

Nucleotide Combinations with No Significant Difference (>0.05)	Num	Total Num	%
The types of trinucleotide combinations contained within tetranucleotide combinations	20		
The number of nucleotide combinations that are consistent with trinucleotide combination	0	4	0
The number of nucleotide combinations that are inconsistent with the trinucleotide combinations	20		
The types of tetranucleotide combinations contained within pentanucleotide combinations	81		
The number of nucleotide combinations that are consistent with tetranucleotide combinations	6	16 **	38
The number of nucleotide combinations that are inconsistent with the tetranucleotide combinations	75		
The types of pentanucleotide combinations contained within hexanucleotide combinations	328		
The number of nucleotide combinations that are consistent with pentanucleotide combinations	42	62	68
The number of nucleotide combinations that are inconsistent with the pentanucleotide combinations	286		
The types of hexanucleotide combinations contained within heptanucleotide combinations	1514		
The number of nucleotide combinations that are consistent with hexanucleotide combinations	233	286 **	81
The number of nucleotide combinations that are inconsistent with the hexanucleotide combinations	1281		

** represents nucleotide combinations with no significant difference, while their reverse complementary sequence combinations exhibit significant differences, with *p*-values for these combinations being only slightly smaller than 0.05.

## Data Availability

The original contributions presented in this study are included in the article/Supplementary Material. Further inquiries can be directed to the corresponding author.

## Supplementary Materials

The following supporting information can be downloaded at https://www.mdpi.com/article/10.3390/plants15172643/s1, Figure S1: Plant evolution analysis based on the proportion of nucleotide combinations. Table S1: Detailed information about the 65 species. Table S2: Proportion of mononucleotide in 64 plant genomes. Table S3: *t*-test. Table S4: *t*-test 2 nucleotide analysis. Table S5: *t*-test 3 nucleotide analysis. Table S6: *t*-test 4 nucleotide analysis. Table S7: *t*-test 5 nucleotide analysis. Table S8: *t*-test 6 nucleotide analysis. Table S9: *t*-test 7 nucleotide analysis. Table S10: *t*-test 8 nucleotide analysis. Table S11: Heatmap of hexanucleotide combinations proportions. Table S12 Classification analysis of A.th 6nc forward and reverse. Supplementary S1: Analysis of nucleotide combination and its reverse complementary sequence. Supplementary S2: rand sequence analysis. Supplementary S3: Analysis program.
