# Supplementary material for: Nucleotide Combination Proportions Across Algae, Monocotyledons and Dicotyledons: Insights into Plant Genome Evolution
Source: Plants (Basel). 2026 Aug 28;15(17):2643. doi: 10.3390/plants15172643 (PMC13567428; doi:10.3390/plants15172643)

Fig. S1 Cluster analysis based on the proportion of nucleotides combinations

Cluster analysis based on the proportion of combinations of three nucleotides

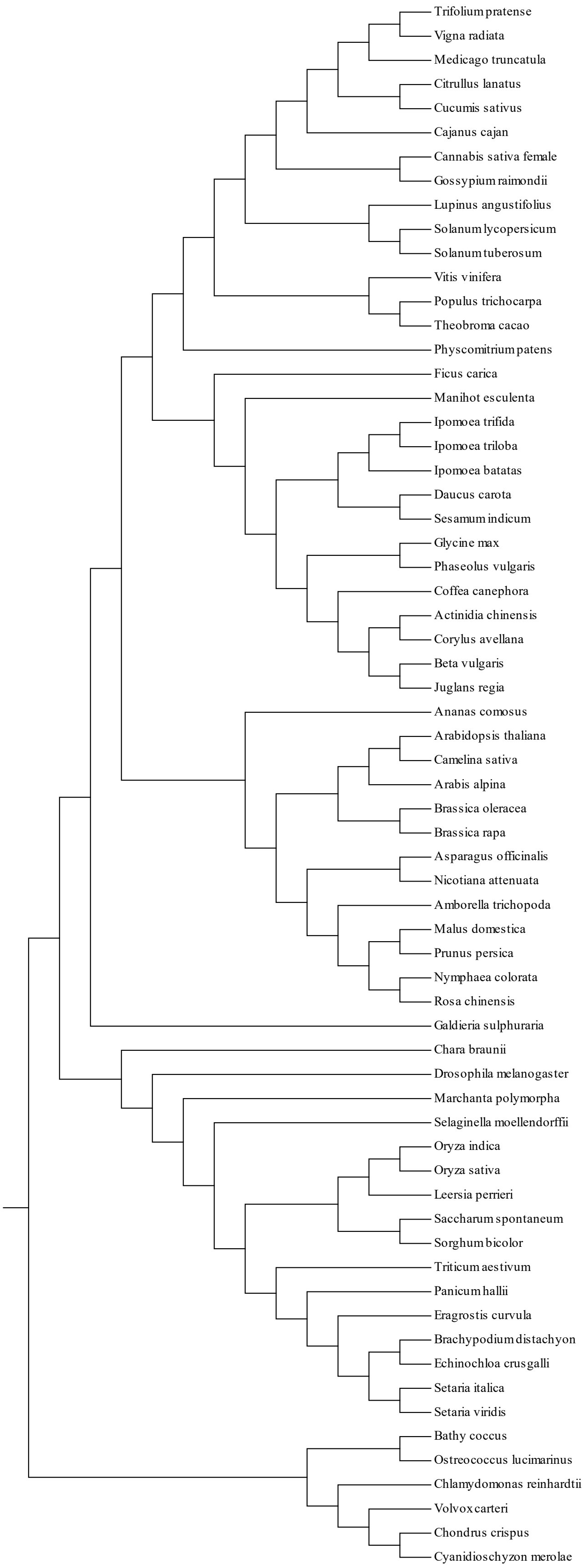

Cluster analysis based on the proportion of combinations of four nucleotides

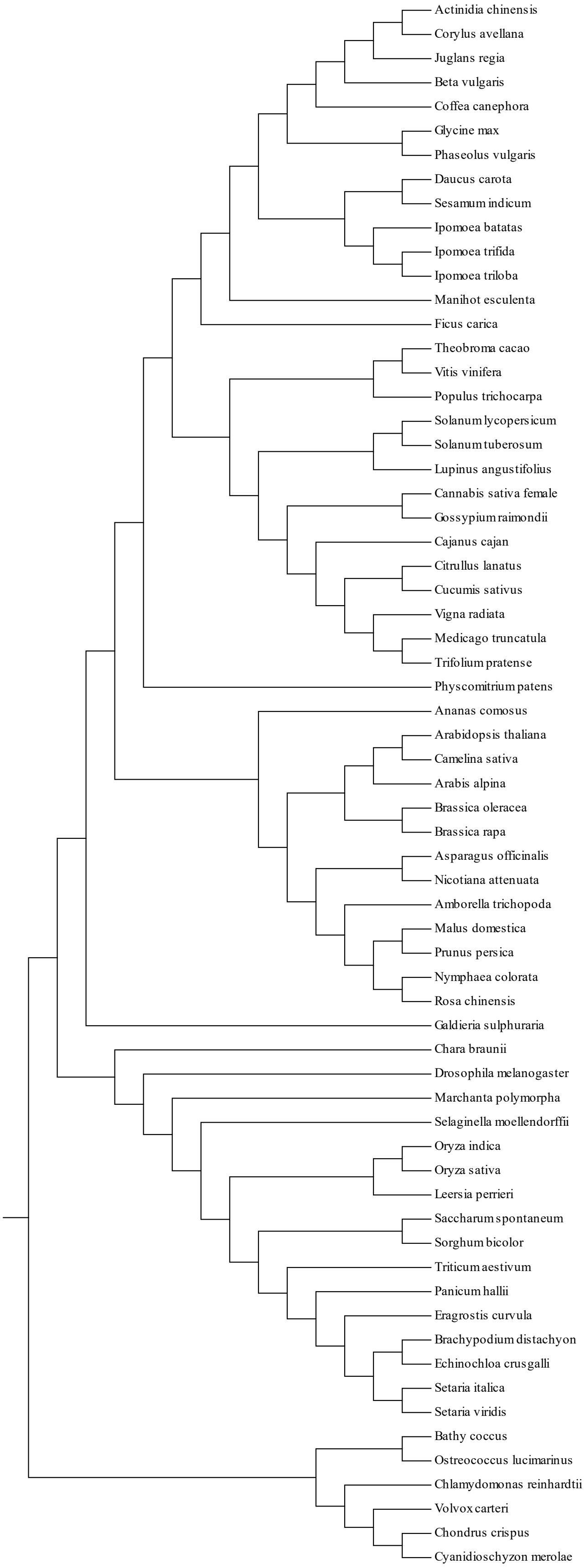

Cluster analysis based on the proportion of combinations of five nucleotides

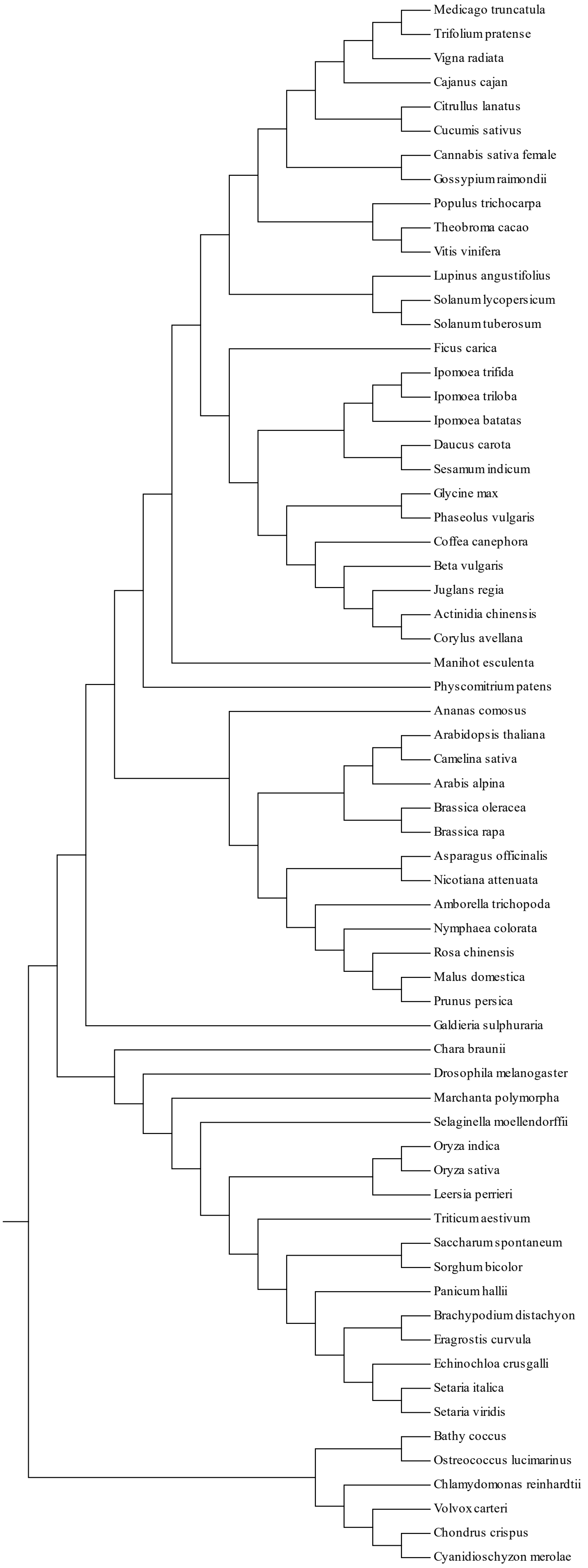

Cluster analysis based on the proportion of combinations of six nucleotides

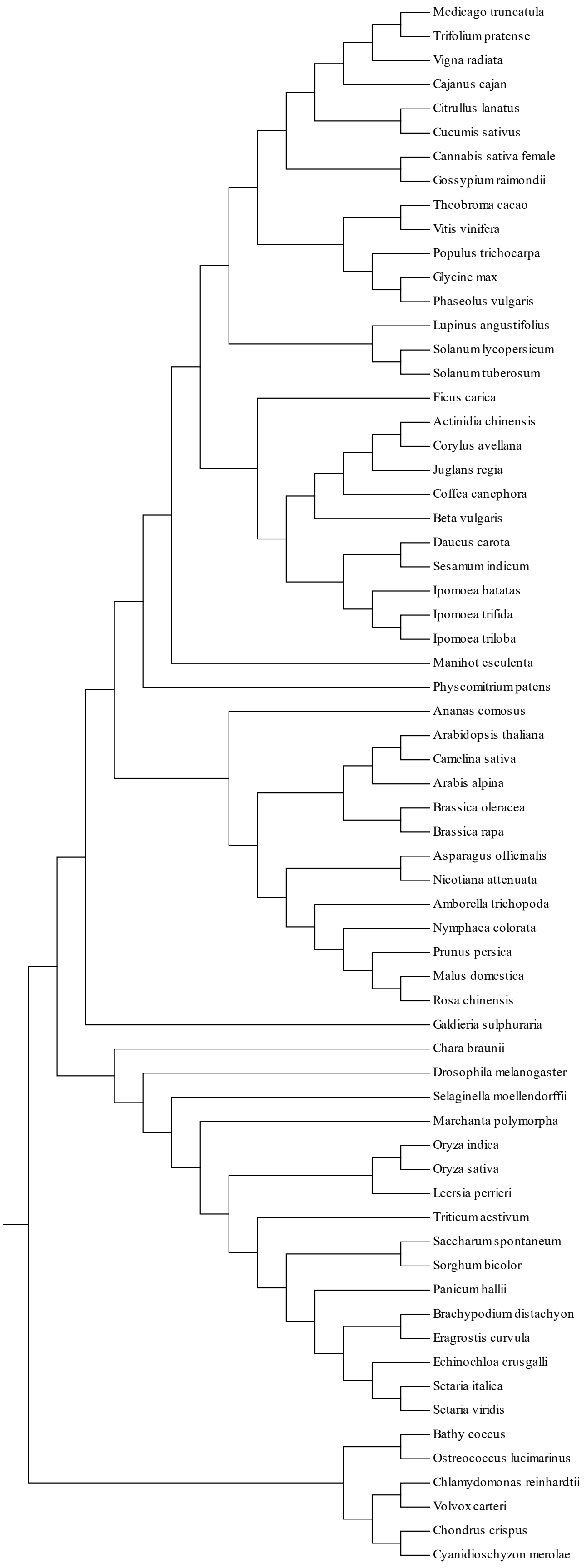

Cluster analysis based on the proportion of combinations of seven nucleotides

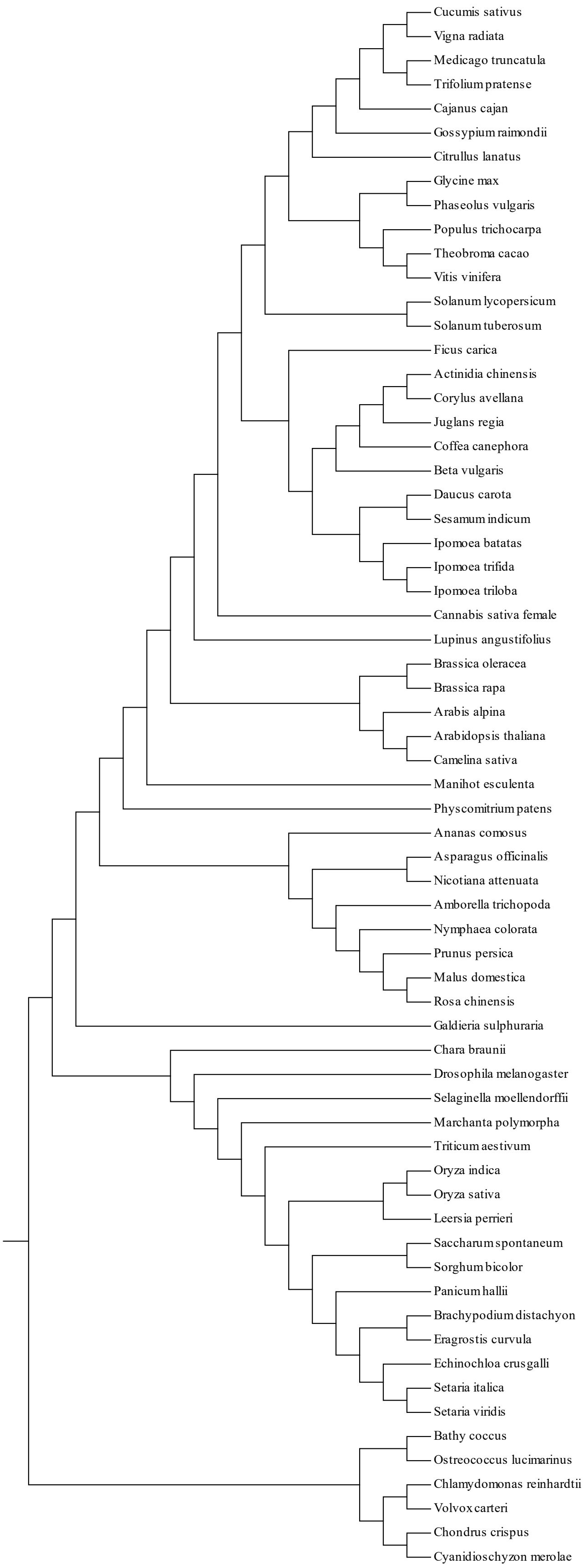

Cluster analysis based on the proportion of combinations of eight nucleotides

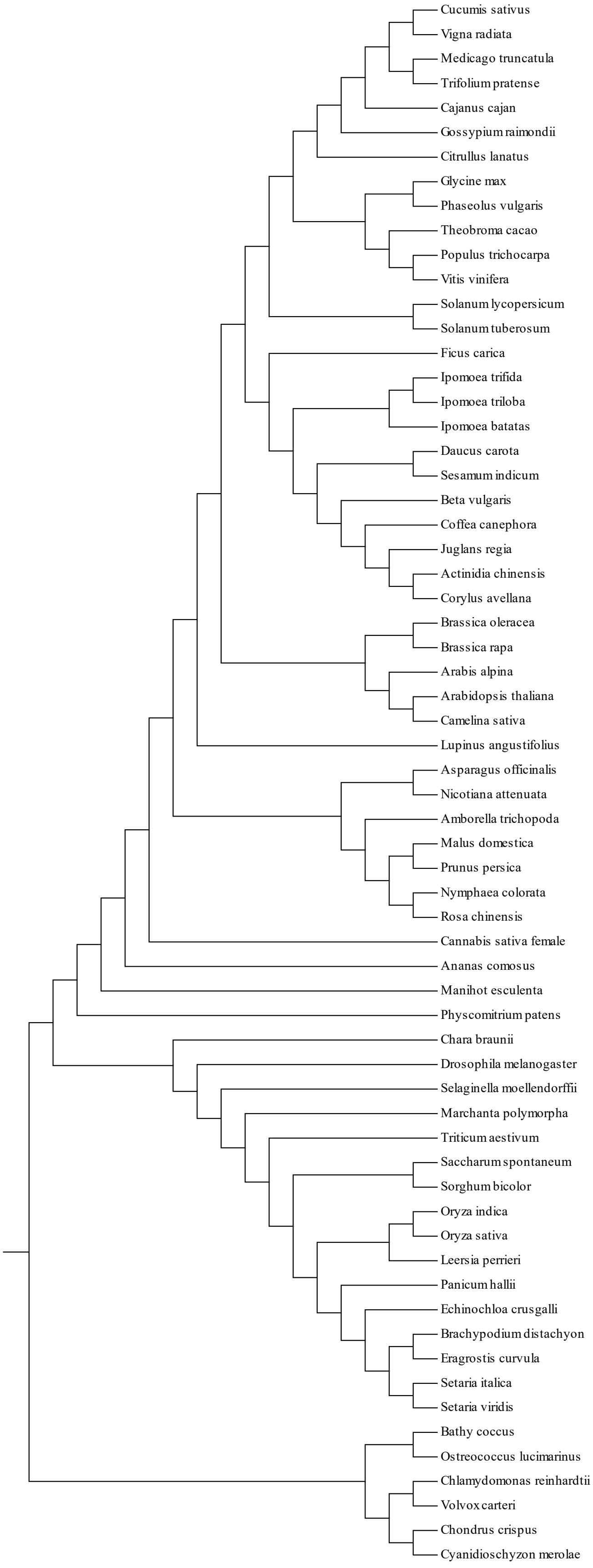

Supplement: Supplementary file 1 [file plants-15-02643-s001.zip › Figure S1 Plant evolution analysis based on the proportion of nucleotide combinations.pdf]
